# Cellular Metabolic Rate Is Influenced by Life-History Traits in Tropical and Temperate Birds

**DOI:** 10.1371/journal.pone.0087349

**Published:** 2014-01-30

**Authors:** Ana Gabriela Jimenez, James Van Brocklyn, Matthew Wortman, Joseph B. Williams

**Affiliations:** 1 Department of Evolution, Ecology and Organismal Biology, Ohio State University, Columbus, Ohio, United States of America; 2 Cancer Institute, University of Cincinnati, Cincinnati, Ohio, United States of America; Clemson University, United States of America

## Abstract

In general, tropical birds have a “slow pace of life,” lower rates of whole-animal metabolism and higher survival rates, than temperate species. A fundamental challenge facing physiological ecologists is the understanding of how variation in life-history at the whole-organism level might be linked to cellular function. Because tropical birds have lower rates of whole-animal metabolism, we hypothesized that cells from tropical species would also have lower rates of cellular metabolism than cells from temperate species of similar body size and common phylogenetic history. We cultured primary dermal fibroblasts from 17 tropical and 17 temperate phylogenetically-paired species of birds in a common nutritive and thermal environment and then examined basal, uncoupled, and non-mitochondrial cellular O_2_ consumption (OCR), proton leak, and anaerobic glycolysis (extracellular acidification rates [ECAR]), using an XF24 Seahorse Analyzer. We found that multiple measures of metabolism in cells from tropical birds were significantly lower than their temperate counterparts. Basal and uncoupled cellular metabolism were 29% and 35% lower in cells from tropical birds, respectively, a decrease closely aligned with differences in whole-animal metabolism between tropical and temperate birds. Proton leak was significantly lower in cells from tropical birds compared with cells from temperate birds. Our results offer compelling evidence that whole-animal metabolism is linked to cellular respiration as a function of an animal’s life-history evolution. These findings are consistent with the idea that natural selection has uniquely fashioned cells of long-lived tropical bird species to have lower rates of metabolism than cells from shorter-lived temperate species.

## Introduction

Life history theory is based on the idea that the schedule and duration of key events in an organism's lifetime are shaped by natural selection to ensure the largest number of surviving offspring in the next generation. These events, juvenile development, age of sexual maturity, age of first reproduction, number of offspring, level of parental investment, and the rate of senescence, are influenced by the abiotic and biotic environment of the organism [1]. Based on [2], variation in life history traits is thought to reflect different allocation patterns of resources to competing life functions, especially growth, body maintenance, and reproduction [3]–. Where adult mortality risk is high, selection should favor animals with rapid growth, and high reproductive output, but at a cost of decreased investment in body maintenance with attendant decreased life span [6]–[10]. Attempts to link life history traits with physiology, particularly the metabolic rate of an organism, have resulted in the notion that life-history variables are constrained within limited ecological space, lying along a “Pace of Life” life-history axis [11]–[13]. Species that have a slow pace of life generally have small clutch sizes, low rates of metabolism, long development times and life spans, whereas species with a fast pace of life have the opposite [10], [12], [14]. Situated at the slow end of the pace of life axis, tropical birds have low annual reproductive output, high annual survival rate, small clutch sizes, slow growth of nestlings, and long post-fledgling dependency on parents [15]–[21], whereas temperate birds tend to cluster more at the fast end of the spectrum, with large clutch sizes and high rates of mortality.

Although it is thought that physiological processes underlie many life-history trade-offs, the physiological mechanisms underlying the diversification of life-histories remain elusive [14], [22], [23]. Past research has often highlighted the fact that similar-sized endotherms, such as a mouse, shrew or bat, can have metabolic rates that vary as much as six-fold, and therefore, natural selection must have adjusted aspects of physiological systems, including organs, tissues, and cells, that influence whole-organism metabolism [24]–[25]. A century after [26] suggested a connection between low metabolic rate and increased longevity, our understanding of functional linkages at the organ, cellular, and molecular level with metabolic rate remain rudimentary [14]. Because the rate of metabolism of an organism integrates numerous aspects of its physiology and links those internal systems with its ecology and life-history, discovering functional linkages between metabolism, at the organismal, cellular, and molecular level, and key attributes of life-history holds considerable promise in advancing our understanding of the connections between physiology and life-history [23], [27]. Evidence of a linkage between a slow pace of life and rate of whole organism metabolism in tropical birds came to light when it was shown that their basal mass-adjusted rate of metabolism was 18% lower than that of temperate birds [28], and that their peak mass-adjusted metabolic rate, as measured by cold-exposure or by exercise, was 30–39% lower than that of temperate birds [29].

The connection between whole-animal metabolic rate and the metabolic rate of cells cultured from animals remains controversial [30]–[32]. One might predict that cells isolated from an organism with a low rate of whole-organism basal metabolism, such as in many tropical birds, would also have a relatively low rate of cellular metabolism. This pattern would support the quantum metabolism theory, which attempts to infer whole organism metabolic rates from the metabolic activities of component cells, posits that cells isolated from animals with low rates of whole-organism metabolism will maintain a low rate of cellular metabolism [30], [33]. However, others argue that metabolically active cells will lose their *in situ* scaling characteristics when they are grown in common nutrient environment, and thus, cultured cells from animals with different rates of mass-specific metabolism will assume a uniform rate of cellular O_2_ consumption [32]. We have tested the hypotheses that cultured cells from tropical birds will have a lower rate of basal, maximal, and non-mitochondrial cellular metabolism, lower rate of proton leak, and lower rates of glycolysis than cultured cells from temperate birds, despite both being cultured in a common thermal and nutritive environment, offering support for the quantum metabolism theory. We used primary dermal fibroblast cell lines from 34 phylogenetically paired species of birds, 17 temperate and 17 tropical, and measured parameters of cellular metabolism. Our results offer compelling evidence that basal, maximal uncoupled, and non-mitochondrial metabolic rate of cells from tropical birds is lower than that of cells from temperate birds, and that cells from tropical birds have a lower proton leak than do cells from temperate species. These data present a unique connection between life-history and rates of metabolism at the cellular level.

## Materials and Methods

### Collection of Birds

Tropical birds were collected by mist-net in the lowland forest around Gamboa, Panama (Latitude: 9.12° and Longitude: −79.72°), and temperate birds were collected in and around Ohio, USA (Latitude: 41.28° and Longitude: 83.1°). All collected birds were adults of unknown age. Temperate birds were collected under an Ohio Division of Wildlife permit number 15–29. All procedures were approved by the Institutional Animal Care and Use Committee of The Ohio State University (protocol IACUC2004A0093) and capture of birds in Panama was accomplished under permit from Panamanian Autoridad Nacional del Ambiente (No. SEX/A-22-12) and Autoridad del Canal de Panamá. All birds were sacrificed by cervical dislocation, prior to collection of skin. Tissues were exported from Panamá the same day as collected under USDA permit 118465 and sent overnight to our lab in Ohio, where they were immediately cultured. Our study was designed to minimize differences in cell physiology as a result of acclimatization of adults because we collected temperate birds during May–July when the average air temperature was at or near ∼23°C, which is only slight below the average air temperature of Panama ∼27°C.

### Establishment of Cell Lines

We evaluated primary dermal fibroblast cell lines from 17 temperate and 17 tropical bird species for their cellular O_2_ consumption capacity and glycolytic capacity. For all species, primary cell cultures were established from the skin of free-living adult birds. Immediately after birds were sacrificed, their feathers were plucked and the exposed skin was washed with anti-microbial soap. We excised a 5×5 mm^2^ piece of skin and placed it into cold complete bird cell culture media (Dulbecco’s modified Eagle medium [DMEM], high-glucose variant [4.5 mg/ml], with sodium pyruvate [110 mg/L], supplemented with 10% heat-inactivated fetal bovine serum, 2% heat-inactivated chicken serum, and antibiotics [100 U/mL pen/strep], containing 10 mM HEPES). Skin from Panamanian birds was sent overnight to our lab at the Ohio State University, where skin was treated exactly as the skin from temperate birds.

We established primary fibroblast cell cultures after the skin was exposed to 0.5% Collagenase B solution overnight. Cells were grown in culture flasks at 37°C in an atmosphere of 5% O_2_ [34]. When cells reached 90% confluence, they were trypsinized (0.25%) and passaged. Seventy five percent of the media was replaced with fresh complete media after day 3 in all flasks, and we split cells into new 75 cm^2^ flasks at day 7–10. After cells grew for another 7 to 10 days, they were harvested and cryopreserved at 10^6^ cells/mL in DMEM supplemented with 40% fetal bovine serum and dimethylsulfoxide (DMSO) at a final concentration of 10%. We stored cells in liquid N_2_ for up to 12 months prior to assessment of their metabolic profile. Previous work from our lab comparing primary dermal fibroblasts across passages and between bird species has found that large scale changes among passages, such as cell size, ratio of non-phospholipids to phospholipids, and the concentrations of the two most common phospholipid classes, tend to be similar among species. Some changes do differ between species, but they occur in such a way that species are becoming more similar to each other [35]. All O_2_ consumption measurements were conducted using cells at passage 2 (P_2_). All cell lines were thawed, resuspended and allowed 5 days to recover from freezing before Oxygen consumption rate (OCR) and Extracellular acidification rate (ECAR) experiments.

### OCR and ECAR Measurements

A Seahorse XF-24 Extracellular flux analyzer was used to measure the rate of O_2_ uptake and CO_2_ efflux in isolated primary dermal fibroblasts from adult birds. Optimization assays were performed prior to experiments to determine the optimal cell seeding density, and optimal concentrations of each compound used for the assay. We seeded 50,000 cells per well per species into XF24 cell culture plates 48 h before experiments. O_2_ consumption rate (OCR) and Extracellular acidification rate (ECAR) was determined following [36], using XF24 FluxPaks (37°C) from Seahorse Bioscience. Measurements of OCR and ECAR were performed after the cells were equilibrated to running media for 1 h. The running medium contained 25 mM glucose and 1 mM sodium pyruvate in all experiments. Because avian plasma typically contains 2–4 times higher glucose concentration than that of mammalian blood, we mixed our running media to contain a glucose concentration that was three times higher than the concentration suggested for running media of mammalian cells [37]. Baseline measurements were made three times prior to injection of 1 µM Oligomycin A, which inhibits ATP synthesis by blocking the proton channel of the F_o_ portion of the ATP synthase. This can be used to distinguish the percentage of O_2_ consumption devoted to ATP synthesis and the oxygen consumption required to overcome the natural proton leak across the inner mitochondrial membrane plus any non-mitochondrial O_2_ consumption. Next, we injected 300 nm FCCP, an ionophore which disrupts the proton gradient, providing a maximal respiratory rate. Finally, we injected 2 µM Antimycin A, a Complex III inhibitor and 100 nm rotenone, a Complex I inhibitor. This combination of chemicals stops mitochondrial respiration and the remaining O_2_ consumption is non-mitochondrial in origin [38]–[41].

When non-mitochondrial O_2_ consumption is subtracted from OCR obtained after the addition of oligomycin, a measurement of proton leak can be estimated. Proton leak is driven by the magnitude of the proton motive force across the inner mitochondrial membrane (Δp) and the rate of proton leak is determined by the inner membrane conductivity to protons at a given Δp [42]. Oligomycin induces a respiratory condition similar to that of state-4 respiration, which increases mitochondrial membrane potential where Δp is maximal and thus proton leak rate is at its maximum [43], [44]. The proton leak values determined in assays using the XF 24 analyzer will be a slight overestimate of the actual leak, and the ATP-linked oxygen consumption rates will be a slight underestimate [45]. Nevertheless, these measurements are useful for examining mitochondrial responses to a given treatment and comparing treatment groups such as birds from tropical and temperate environments.

After O_2_ consumption assays were performed, we counted the final concentration of cells in each well and normalized all rates to a total of 50,000 cells as a standard. We additionally measured cell diameters at P2 for each species to determine if fibroblast cells from different species were of different size. To measure cell diameter, we took images of rounded cells after trypsinization for all species using a CCD digital camera through a 10x power objective, and measured cell size using Labkit Image J. We measured 30 cells per individual to obtain mean values. For OCR measurements, we used the third basal measurement made by the XF24, for maximal metabolic rate, we took the OCR value after injection of FCCP, and non-mitochondrial respiration was the lowest measurement after the injection with Antimycin A and rotenone. The spare respiratory capacity was calculated as the maximal metabolic rate divided by the basal respiratory rate with the minimum non-mitochondrial respiration subtracted from both. Spare respiratory capacity provides an idea of a cell’s maximum ATP production, therefore, cells with higher capacity have greater ability to respond to stress [37]–[41]. For ECAR measurements, we looked at basal ECAR values as the third basal measurement prior to any injections (Fig. S1).

### Statistics

We used a Shapiro-Wilk test to check for normality in our data. To determine statistical difference between tropical and temperate pairs, we used mean values for each parameter as a function of environment and applied a Wilcoxon signed ranks test [46]. We performed statistical tests using SPSS 19.0, with α = 0.05.

## Results

### Paired Comparisons

In comparative biology, most investigators attempt to avoid statistical problems associated with the lack of independence of data from closely related species by using one or more methods that take into account phylogenetic relatedness [47]–[49]. To use these methods, one must test hypotheses by embedding data in another hypothesis, the phylogeny, which may or may not be accurate, and then constructing contrasts that can be employed in analyses [50], [51], [20]. By comparing cells from sister species, one tropical and one temperate, that have diverged relatively recently on a geological time scale, we employ here a powerful comparative approach that reduces problems of non-independence of data, and problems associated with differences in body size because closely related species are often similar in body mass [52]. A priori, we situated our paired comparisons within a phylogenetic tree based on [52]–[56] (Fig. S2). Our paired comparisons include a diverse range of avian orders within the avian phylogeny and include non-passerines and passerines. Mean body masses for our 34 species of tropical and temperate birds ranged from 2.9 g to 327 g, a 100-fold range across species (Table 1).

**Table 1:** Common name, species name, sample size used for all OCR and ECAR measurements and mean body mass for each phylogenetically-paired temperate and tropical species.

Common name	Species	Environment	N	Bodymass (g)	Common name	Species	Environment	N	Body mass (g)
Ruby-throatedHummingbird	*Archilochus colubris*	Temperate	3	2.9	Rufous-tailed hummingbird	*Amazilia tzacatl*	Tropical	7	4.63
Yellow warbler	*Setophaga petechia aestiva*	Temperate	3	9.94	Mangrove warbler	*Setophaga petechia aequatorialis*	Tropical	1	9.05
Tree swallow	*Tachycineta bicolor*	Temperate	3	22.38	Southern rough-winged swallow	*Stelgidopteryx serripennis*	Tropical	3	10.75
Eastern wood pewee	*Contopus virens*	Temperate	3	14.21	Toddy flycatcher	*Todirostrum chrysocrotaphum*	Tropical	2	6.25
Red-eyed vireo	*Vireo olivaceus*	Temperate	3	16.18	Yellow-green vireo	*Vireo flavoviridis*	Tropical	4	15.18
House finch	*Haemorhous mexicanus*	Temperate	4	20.56	Thick-billed euphonia	*Euphonia laniirostris*	Tropical	3	12.32
Carolina wren	*Thryothorus ludovicianus*	Temperate	4	22.6	Buff-breasted wren	*Cantorchilus leucotis*	Tropical	4	18.69
Song sparrow	*Melospiza melodia*	Temperate	4	20.46	Black-striped sparrow	*Arremonops conirostris*	Tropical	3	36.2
Northern cardinal	*Cardinalis cardinalis*	Temperate	3	38.89	Red throated tanager	*Habia fuscicauda*	Tropical	3	37.65
Red-winged blackbird	*Agelaius phoeniceus*	Temperate	6	69.63	Red-breasted blackbird	*Sturnella militaris*	Tropical	5	41.75
Gray catbird	*Dumetella carolinensis*	Temperate	3	35.42	Tropical Mockingbird	*Mimus gilvus*	Tropical	3	58.4
Eastern whip-poor-will	*Antrostomus vociferus*	Temperate	3	51.68	Common pauraque	*Nyctidromus albicollis*	Tropical	6	55.17
American robin	*Turdus migratorius*	Temperate	5	78.2	Clay-colored Robin	*Turdus grayi*	Tropical	4	79.5
Red-bellied woodpecker	*Melanerpes carolinus*	Temperate	3	73.2	Red-crowned woodpecker	*Melanerpes rubricapillus*	Tropical	4	55.9
Mourning dove	*Zenaida macroura*	Temperate	3	124.53	White-tipped dove	*Leptotila verreauxi*	Tropical	4	161
Common grackle	*Quiscalus quiscula*	Temperate	3	128.44	Great-tailed grackle	*Quiscalus mexicanus*	Tropical	3	222
Killdeer	*Charadrius vociferus*	Temperate	3	92.1	Southern Lapwing	*Vanelus chilensis*	Tropical	2	327

Pairs are listed on the same row and their environment is indicated on the table.

### Cell Size

We measured the diameter of cells for each species at passage 2 and found no significant differences between cell size across species. (Wilcoxson test, P>0.2; Fig. S3).

### Basal Cellular O_2_ Consumption (OCR)

**Figure 1:**
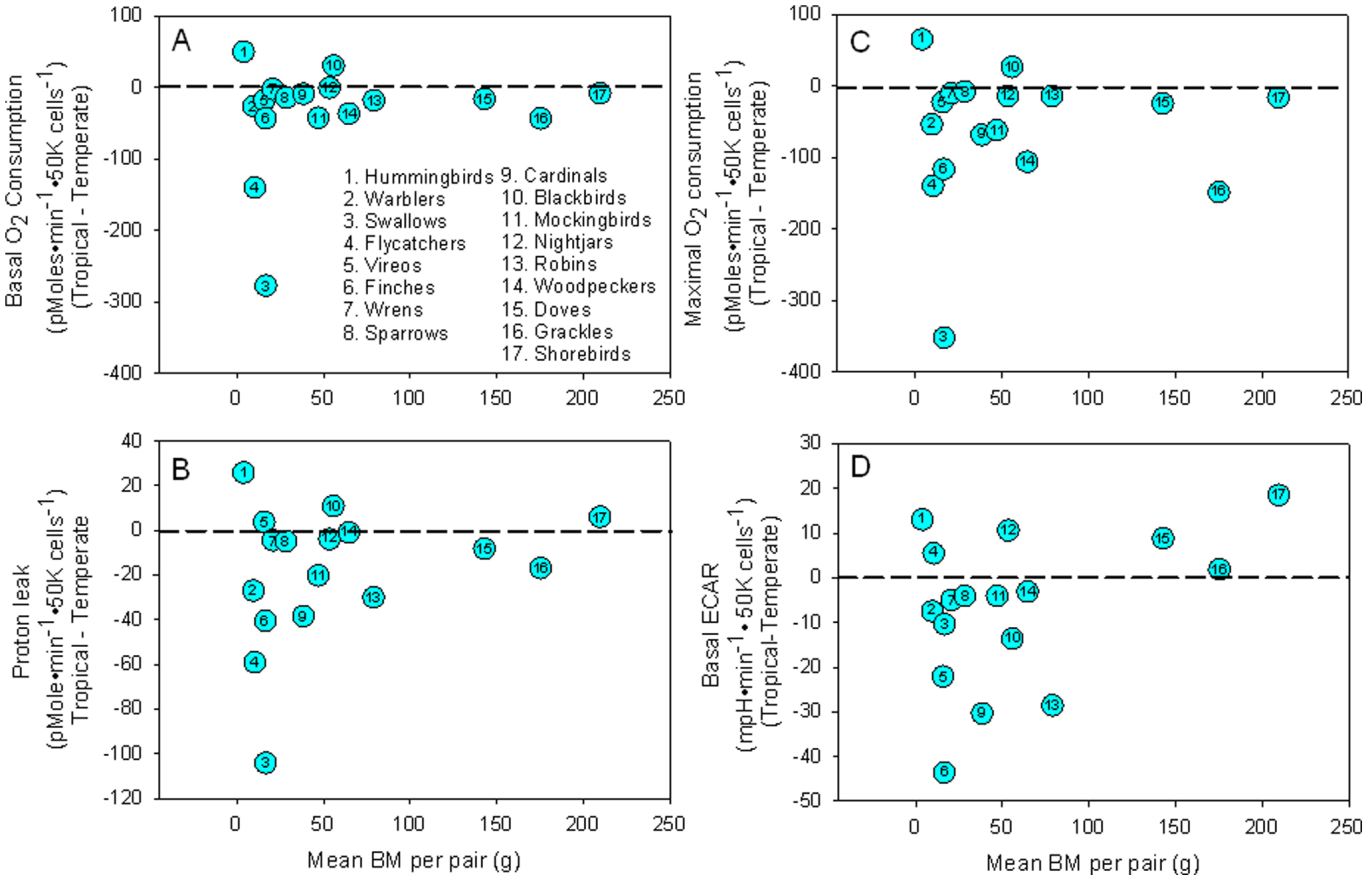
Differences between mean basal and maximal respiratory rate in tropical and temperate species as they relate to mean body mass for both tropical and temperate species (A) represents basal rates, (B) represents proton leak rates, (C) represents uncoupled rates, and (D) represents basal glycolytic rates, where the dashed line represents the limit differences between tropical and temperate species. The difference between the two rates shows a predominantly negative rate for most species included, indicating that tropical values were generally lower than temperate values. Sample size is the same as Table 1.

Normalized basal cell O_2_ consumption was significantly lower in cells from tropical birds compared with cells from temperate birds (Wilcoxson test; P = 0.015) (Table 2, Fig. 1A). Basal cell O_2_ consumption was on average 29% lower in cells from species of tropical birds compared with cells from temperate counterparts.

**Table 2:** Cellular consumption rate (OCR) parameters for each species, including basal, maximum, non-mitochondrial metabolic rates and spare respiratory capacity.

Common name	Basal MR (pMole⋅min^−1^⋅50 K cells^−1^)	Maximal MR (pMole⋅min^−1^⋅50 K cells^−1^)	Non-mito respiration (pMole⋅min^−1^⋅50 K cells^−1^)	Spare respiratory capacity (%)	Common name	Basal MR (pMole⋅min^−1^⋅50 K cells^−1^)	Maximal MR (pMole⋅min^−1^⋅50 K cells^−1^)	Non-mito respiration (pMole⋅min^−1^⋅50 K cells^−1^)	Spare respiratory capacity (%)
Ruby-throated Hummingbird	31.30±5.90	50.47±8.89	20.18±5.11	273±92.29	Rufous-tailed hummingbird	81.09±5.30	116.29±9.74	54.44±5.08	749.98±327.59
Yellow warbler	106.42±17.55	162.55±22.79	69.78±11.95	539.38±161.29	Mangrove warbler	80.11	109.55	51.62	152.35
Tree swallow	398.72±94.16	523.85±123.15*	176±41.46	536.23±83.12	Rough-winged swallow	121.29±26.36	172.40±31.18	80.20±19.37	1118.08±331.18
Eastern wood pewee	188.16±52.98	221.13±54.09	105.05±31.55	947.21±445.77	Toddy flycatcher	47.60±5.91	82.22±9.25	39.02±5.41	816.86±48.40
Red-eyed vireo	323.96±60.96	339.84±56.82	152.93±25.26	361.40±129.96	Yellow-green vireo	305.74±39.72	317.91±53.83	154.57±26.30	277.17±73.18
House finch	92.19±28.61	187.93±25.03	85.41±10.87	374.26±51.21	Thick-billed euphonia	48.87±13.53	72.16±13.72	23.76±5.36	158.52±51.55
Carolina wren	50.36±7.09	92.87±15.18	24.64±6.11	183.40±36.36	Buff-breasted wren	47.84±4.71	83.11±6.78	19.99±4.19	164.96±31.10
Song sparrow	86.76±15.69	111.69±12.02	41.81±5.97	187.06±27.70	Black-striped sparrow	72.15±16.58	104.68±20.76	30.55±6.27	137.23±26.43
Northern cardinal	76.42±17.74	141.97±26.01	51.94±25.51	120.44±14.72	Red throated tanager	67.22±9.90	74.22±10.82	27.77±3.58	83.56±17.41
Red-winged blackbird	63.83±10.02	100.81±11.49	85.50±51.49	528.14±109.63	Red-breasted blackbird	94.04±15.99	127.81±21.29	57.64±14.50	294.25±85.05
Gray catbird	141.46±12.60	193.02±36.41	76.99±12.48	223.05±58.35	Tropical Mockingbird	98.89±6.74	131.83±18.92	51.69±4.37	104.28±20.12
Eastern whip-poor-will	71.94±10.13	113.06±16.02	39.37±8.70	298.85±54.46	Common pauraque	71.32±10.01	99.50±11.71	35.50±4.90	512.71±142.36
American robin	92.67±36.46	117.31±35.89	59.59±27.82	170.29±19.55	Clay-colored Robin	74.44±9.61	103.78±13.33	41.16±6.89	248.17±48.83
Red-bellied woodpecker	138.28±23.04	241.68±45.68	50.74±15.14	1304.63±496.91	Red-crowned woodpecker	101.59±18.21	136.21±33.78	48.48±15.71	704.93±424.92
Mourning dove	45.39±7.82	76.94±4.60	26.75±4.18	217.63±33.15	White-tipped dove	29.34±6.51	52.85±9.42	15.19±4.87	502.83±118.65
Common grackle	151.75±91.60	261.61±158.40	76.80±64.33	395.79±125.33	Great-tailed grackle	108.16±20.25	113.51±13.80	39.21±7.14	296.88±66.15
Killdeer	49.17±10.23	82.61±17.75	28.08±5.59	211.67±44.80	Southern Lapwing	41.02±5.84	67.30±17.01	27.15±8.90	252.68±81.44

Values represent mean ± SEM. Sample size is the same as Table 1.

### Uncoupled Cellular O_2_ Consumption (OCR)

Uncoupled O_2_ consumption of cells from species of tropical birds was significantly lower than cells from temperate species (Wilcoxon test; P<0.006; Table 2, Fig. 1C). Spare respiratory capacity was similar among tropical and temperate birds (Wilcoxon test; P>0.5; Table 2).

### Proton Leak (OCR)

Proton leak was significantly lower in cells from tropical birds compared with cells from temperate birds (Fig. 1B; Wilcoxon test; P<0.02).

### Non-mitochondrial Respiration (OCR)

Cells of tropical birds had a significantly lower non-mitochondrial O_2_ consumption than did cells from temperate birds (Wilcoxon test; P<0.004: Table 2), a 32% decrease.

### Basal Extracellular Acidification Rate (ECAR)

Anaerobic glycolysis can be estimated by the flux of protons from the cell (Brand, 1990). Anaerobic breakdown of glucose produces an excess of protons, resulting in acidification of the medium surrounding the cells. Basal ECARs were not statistically different between cells from tropical and temperate birds (Wilcoxon test, P>0.05; Table 3; Fig. 1D).

**Table 3:** Basal extracellular acidification rate (ECAR) measurements used to estimate anaerobic glycolysis in cells from temperate and tropical birds.

Common name	Basal ECAR(mpH⋅min^−1^⋅50 K cells^−1^)	Common name	Basal ECAR(mpH⋅min^−1^⋅50 K cells^−1^)
Ruby-throated Hummingbird	18.89±2.56	Rufous-tailed hummingbird	31.90±3.59
Yellow warbler	25.53±4.31	Mangrove warbler	18.05
Tree swallow	39.55±9.71	Rough-winged swallow	29.16±7.02
Eastern wood pewee	48.64±15.40	Toddy flycatcher	54.10±20.31
Red-eyed vireo	53.29±8.64	Yellow-green vireo	31.24±4.66
House finch	67.80±17.49	Thick-billed euphonia	24.24±3.39
Carolina wren	20.68±2.33	Buff-breasted wren	15.73±1.72
Song sparrow	22.23±3.83	Black-striped sparrow	18.13±2.73
Northern cardinal	43.87±14.84	Red throated tanager	13.51±1.81
Red-winged blackbird	53.48±7.67	Red-breasted blackbird	39.86±9.78
Gray catbird	16.94±1.16	Tropical Mockingbird	12.85±0.74
Eastern whippoorwill	20.42±5.09	Common pauraque	31.01±4.36
American robin	47.98±31.79	Clay-colored Robin	19.47±2.63
Red-bellied woodpecker	56.56±10.81	Red-crowned woodpecker	53.48±12.49
Mourning dove	12.66±1.96	White-tipped dove	21.49±1.02
Common grackle	28.35±8.64	Great-tailed grackle	30.22±3.54
Killdeer	13.97±3.30	Southern Lapwing	32.51±7.53

Values represent mean ± SEM. Sample size is the same as Table 1.

## Discussion

We have cultured primary dermal fibroblasts in a common nutritive and thermal environment from phylogenetically-paired tropical and temperate species of birds to test the idea that differences in life history are accompanied by intrinsic differences in cellular metabolism. Previous work suggested that, when compared with temperate species, tropical birds have an 18% lower basal metabolic rate and a >30% lower peak metabolic rate, for the latter, as elicited by cold-exposure or flight wheel, [28], [29]. Our paired comparisons revealed that basal cellular metabolic rate was 29% lower in tropical birds and maximal uncoupled cellular metabolic rate was 35% lower in tropical species compared with temperate, consistent with their lower whole-animal metabolic rate (Fig. 1A, B) [28], [29]. We also found a significant decrease in non-mitochondrial respiration and proton leak in cells from tropical birds (Fig. 1C; Table 2). These results suggest that birds of common phylogenetic lineage, but differing life-histories, have cells with intrinsic physiological properties that are consistent with the metabolic signature of the whole-animal.

Mitochondria typically show a leak of protons back across inner mitochondrial membrane, which results in oxidative phosphorylation not being completely coupled to electron flow [57], [58]. Proton leak is energetically costly for animals; it accounts for 20% of respiration in rat hepatocytes and 50% of respiratory rate in rat muscle [59], [60]. Explanations for why animals would evolve mechanisms that promote a leak of protons across the inner mitochondrial membrane include a role in thermogenesis, and a role in attenuation of ROS production [60]. Because information on mitochondrial proton leak has been gathered from isolated mitochondria, a preparation that exposes them to atmospheric O_2_ tensions (21%) rather than the lower O_2_ tensions found within a living cell (3–5%), conclusions should be regarded as tentative [58]. Advancements in our understanding of mitochondrial function and proton leak in particular, will likely come by work on intact cells rather than isolated mitochondria. If in endotherms, proton leak is a component of thermogenesis, then cells from tropical birds with their reduced basal rate of whole-animal metabolism might, by hypothesis, have reduced proton leak, whereas cells from temperate birds with their higher BMR and greater need for thermogenesis might be expected to have greater proton leak.

Because differences in cellular metabolism are directly related to mitochondrial function, differences in mitochondrial density or morphology may account for the differences in cellular metabolic rates that we have found. Fibroblasts from tropical birds had 26% less mitochondrial lipid than did cells from tropical species, and cells from tropical birds had 26% less cardiolipin [36]. We infer from these data that fibroblasts of tropical birds have either fewer mitochondria, or less inner mitochondrial membrane per mitochondrion [61]. Found exclusively in the inner mitochondrial membrane, cardiolipin is required for maintenance of the electricochemical potential across the inner membrane and decreases in cardiolipin content can result in a decrease in the activity of Complex I [61], [62]. Previous work that has found an increase in cardiolipin in cells to be related to an increase in number of mitochondria [63], thus, we assume that temperate birds have a higher number of mitochondria, which drives their higher cellular metabolic rates, as compared with tropical species.

Some colleagues might suggest that it is intuitive that animals with lower whole-organism basal metabolism, like tropical birds, would also have cells with a lower intrinsic rate of metabolism when those cells are grown in culture. However, the connection between whole-organism physiology and intrinsic physiological function of isolated cells in a culture environment has been controversial, especially in the medical community. The idea that intrinsic rates of cell metabolism should mirror whole-organism metabolism has its roots in experiments which related metabolism of slices of liver, or a heterogeneous collection of hepatocytes, to animals of different body size: tissue slices or liver cells from large mammals, with their lower mass-adjusted whole-organism metabolism, had lower O_2_ consumption than did tissue slices from small mammals [44], [64]. In contradistinction, there now exists some evidence that when cells are isolated from an animal, and cultured, that these cells assume a common rate of metabolism. [65] first proposed that the metabolic rate of cells in animals was dictated by the rate of delivery of nutrients from their circulatory system. From a review of the literature, [66] concluded that differences in mass-adjusted metabolism of mammals of different body size did not reside in cellular function but at higher levels of physiological organization. Recently, [32] argued that metabolic scaling followed a fractal-like design at all levels of animal organization, and predicted that the metabolic rate of cultured mammalian cells would be uniform and independent of animal body mass. An experimental test of this hypothesis found that primary dermal fibroblasts for mammals of different body sizes assumed a uniform rate of cellular O_2_ consumption [67]. The fact that we have shown significant differences in the intrinsic rate of cellular metabolism between tropical and temperate birds how evolution of life history traits could be linked with differences in cell function.

Fibroblast cells are widely used as a tool to study a range of physiological, medical, and pathological processes [67]–[84]. However, few researchers have compared the pattern of cell metabolism of this cell type with other cell types in the body of the same individual. Whole-animal metabolism encompasses the summation of each organ’s metabolic rate in the body. Muscle tissue contributes more to basal metabolic rate than other organs such as liver, brain, and kidneys even though these latter tissues are more metabolically active on a mass-specific basis [64], [79]. However, muscle makes up the largest fraction of total body mass, so its contribution to resting metabolic rate is higher than that of other tissues [85]. Connective tissue has a small contribution to basal metabolic rate in animals, thus, one might question whether fibroblasts represent a model system that characterizes the metabolic profile of other tissues like muscle. Dermal fibroblasts are responsible for generating connective tissue and are involved in wound healing [86] but generally this cell type is thought to be metabolically inactive until it is required at the site of tissue damage. To address this concern, we have cultured muscle satellite cells and fibroblasts from coturnix quail chicks selected for fast (analogous to temperate birds) or slow growth (like tropical birds). Parallels in the metabolism of different cell types are perhaps not intuitive, since they perform such different functions. However, we found that the metabolic signature between myoblast cells and fibroblasts was identical, depicting a lower basal and maximal OCR for the slow-growth line compared with the fast-growth line (Jimenez et al. in review). Our results suggest that isolated fibroblasts and myoblasts had the same metabolic profile, which suggests that fibroblasts are a representative cell system for the organism. Additionally, cellular metabolic differences could also occur for other organs, and since tropical birds generally have smaller metabolically active organs [87], then both cellular metabolic rates and organ masses are likely to alter organismal metabolic rate differences.

Life-history theory suggests that lower extrinsic mortality results in low reproductive effort and more energy allocation to self-maintenance in the tropics [1], thus, increasing longevity. We think that part of the increase in self-maintenance of tropical birds manifests itself in a slower pace of life, reflected in decreased BMR and PMR, smaller metabolically active organs, and lower cellular metabolic rate [28], [29], [87]: collectively all of these are aspects of the birds’ physiology which positively correlate with increased survival. Physiological and fitness costs of particular life-history choices are dependent on environmental conditions [10], and it is difficult to separate the influences of climate per se and life-history attributes. Our study was designed to minimize differences in cell physiology as a result of acclimatization because we collected temperate birds during May-July when the average air temperature was ∼23°C, and the average air temperature of Panama was ∼27°C. Thus, we think that differences at the cellular level between tropical and temperate birds are likely the result of intrinsic differences caused by natural selection. Our study represents a major advancement in our understanding of how underlying physiological mechanisms at the cellular level are linked to life history [10].

In summary, we have isolated primary dermal fibroblasts from temperate and tropical birds with differing life-history traits and have cultured these cells under the same conditions through two passages. We found that intrinsic rate of metabolism of cells from tropical birds was significantly lower than was cells from temperate birds. Tropical birds had lower basal and peak whole-animal metabolic rates, and these rates appear to be reflected by metabolic rates of individual cells. Physiological differences at the cellular level such as reduction in mitochondrial density and/or efficiency, has apparently allowed tropical and temperate birds to have different cellular metabolic rates. Our study links aspects of cellular function to the life-history evolution of tropical and temperate bird species.

## Supporting Information

Figure S1 **Example of a typical metabolic profile illustrating OCR responses for avian dermal fibroblasts when exposed to metabolic inhibitors.**
(TIF)

Figure S2 **Phylogenetic tree of 34 tropical and temperate bird species.** Phylogenetic relationships are based on information assembled by (Johnson and Sorenson, 1999; Klicka et al., 2000; Yuri and Mindell, 2002; Boyd, 2011; Jetz et al 2012). Branch lengths were derived from Sibley and Alquist (1990) and shown in units of difference in melting temperatures of bonded DNA strands of different species. Tropical species are designated in red lettering, and temperate species in black lettering.
(TIFF)

Figure S3 **Cell size comparison across species.** Images were taken using an Olympus light microscope at 10 x after trypsinazation at passage 2 (P2). Cell diameters were measured using Image J. Values represent mean ± SEM. (p>0.2).
(TIF)
