# Supplementary figures and images for: Cellular Metabolic Rate Is Influenced by Life-History Traits in Tropical and Temperate Birds
Source: PLoS One. 2014 Jan 30;9(1):e87349. doi: 10.1371/journal.pone.0087349 (PMC3907555; doi:10.1371/journal.pone.0087349)

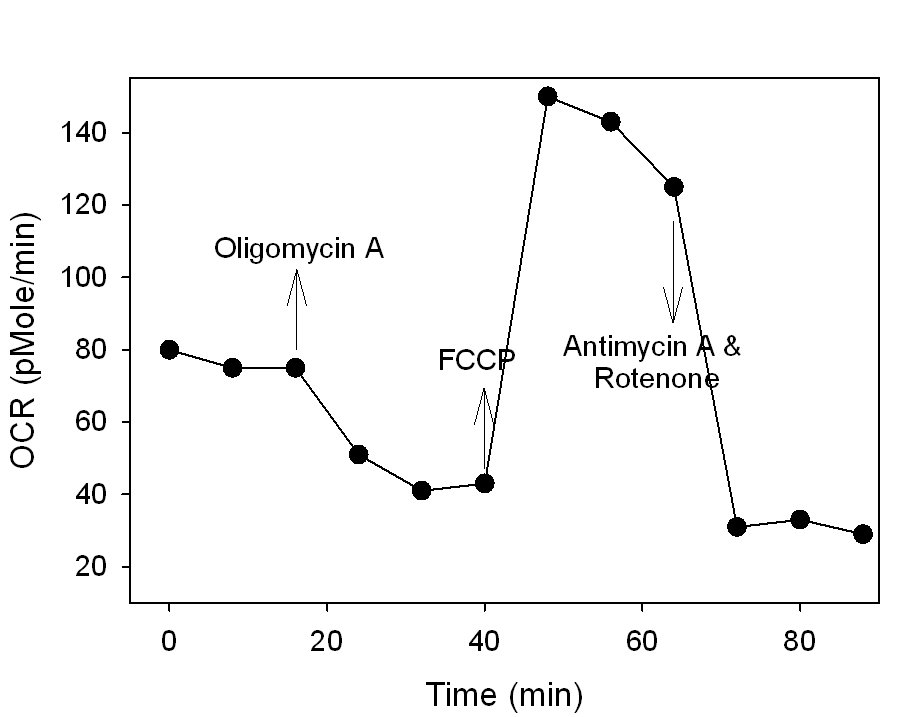

Supplement: Figure S1 — Example of a typical metabolic profile illustrating OCR responses for avian dermal fibroblasts when exposed to metabolic inhibitors. (TIF) [file pone.0087349.s001.tif]

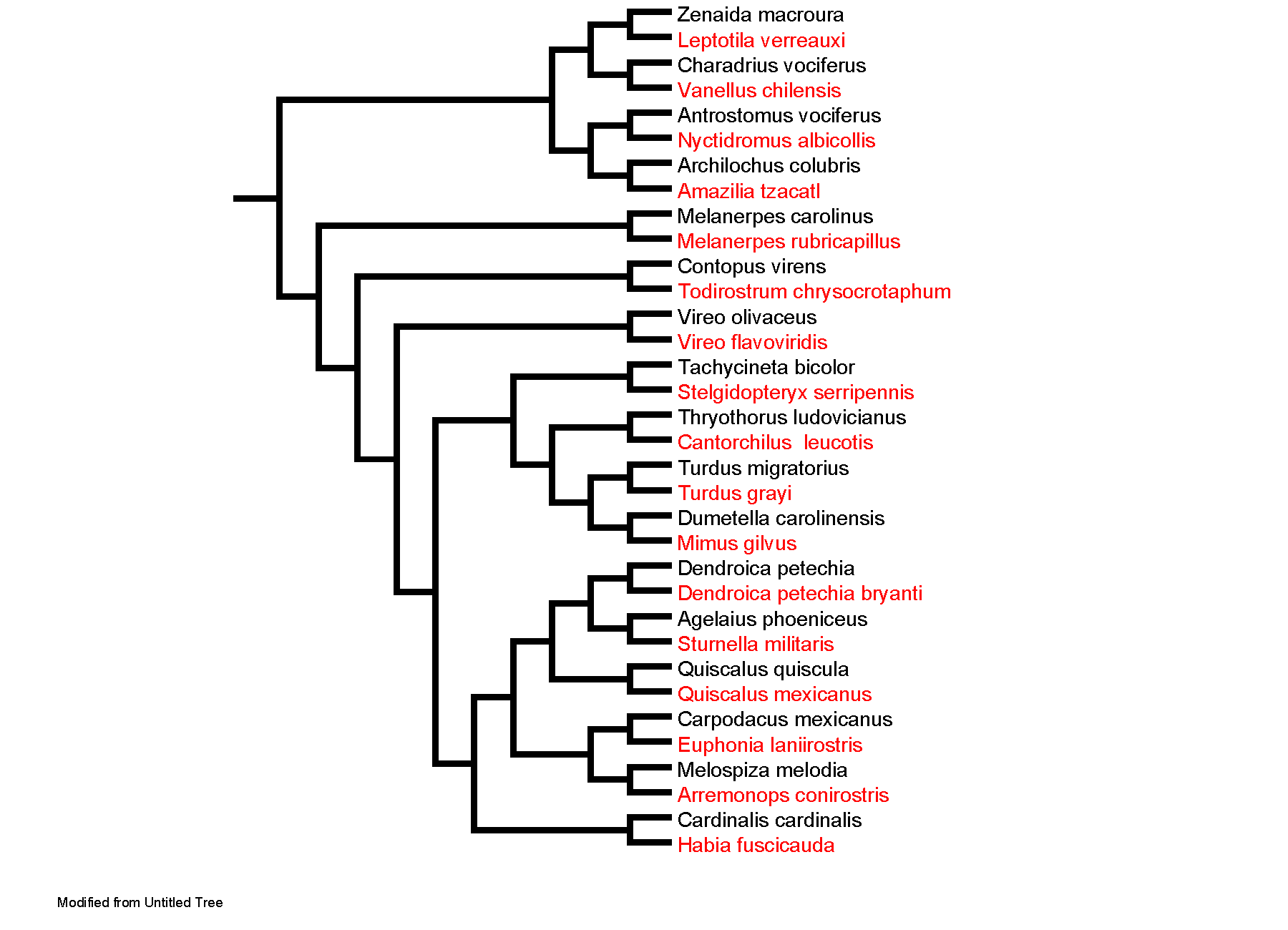

Supplement: Figure S2 — Phylogenetic tree of 34 tropical and temperate bird species. Phylogenetic relationships are based on information assembled by (Johnson and Sorenson, 1999; Klicka et al., 2000; Yuri and Mindell, 2002; Boyd, 2011; Jetz et al 2012). Branch lengths were derived from Sibley and Alquist (1990) and shown in units of difference in melting temperatures of bonded DNA strands of different species. Tropical species are designated in red lettering, and temperate species in black lettering. (TIFF) [file pone.0087349.s002.tiff]

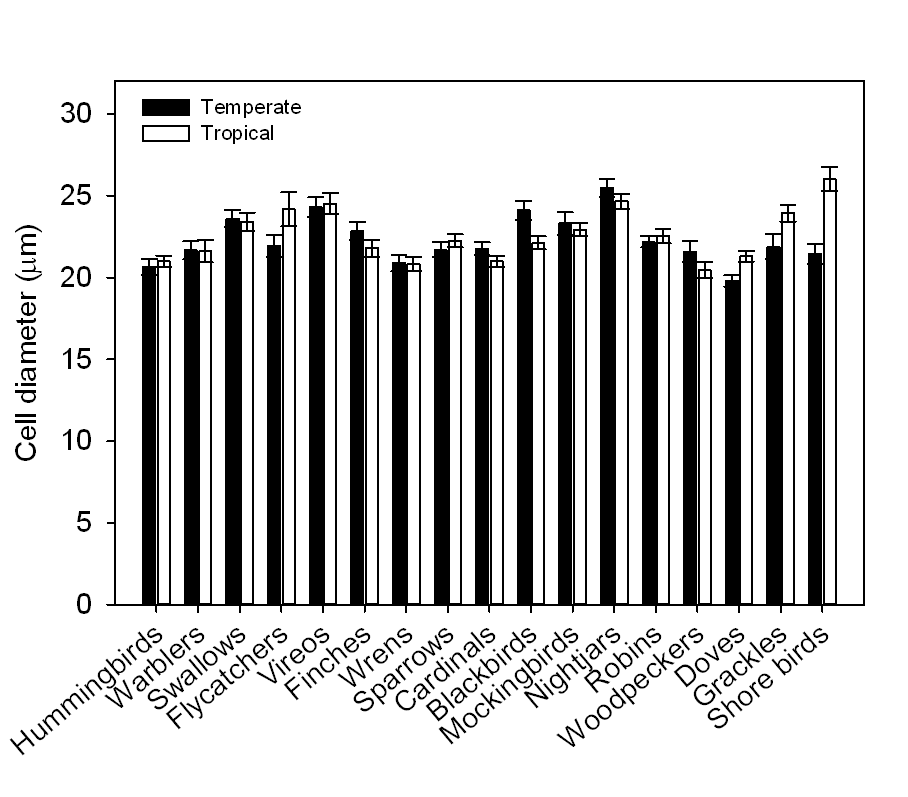

Supplement: Figure S3 — Cell size comparison across species. Images were taken using an Olympus light microscope at 10 x after trypsinazation at passage 2 (P2). Cell diameters were measured using Image J. Values represent mean ± SEM. (p>0.2). (TIF) [file pone.0087349.s003.tif]
